# Minimizing risk of customized titanium mesh exposures – a retrospective analysis

**DOI:** 10.1186/s12903-020-1023-y

**Published:** 2020-02-03

**Authors:** Amely Hartmann, Marcus Seiler

**Affiliations:** 1 Private Practitioner, Affiliate to the Department of Oral and Maxillofacial Surgery, University Medical Centre of the Johannes Gutenberg University of Mainz, Augustusplatz 2, 55131 Mainz, Germany; 2 Department Head, Private Dental Practice, Echterdinger Str. 7, 70794 Filderstadt, Germany

**Keywords:** Customized titanium mesh, Digital dentistry, Mesh exposure, Risk parameters, Platelet rich fibrin

## Abstract

**Background:**

Recommendations for soft tissue management associated with customized bone regeneration should be developed. The aim of this study was to evaluate a new protocol for customized bone augmentation in a digital workflow.

**Methods:**

The investigators implemented a treatment of three-dimensional bone defects based on a customized titanium mesh (Yxoss CBR®, ReOSS, Filderstadt, Germany). Patients and augmentation sites were retrospectively analysed focussing on defect regions, demographic factors, healing difficulties and potential risk factors. An exposure rate was investigated concerning surgical splint application, A®- PRF and flap design.

**Results:**

In total, 98 implants could be placed. Yxoss CBR® was removed after mean time of 6.53 ± 2.7 months. Flap design was performed as full flap preparation (27.9%), full flap and periosteal incision (39.7%), periosteal incision (1.5%), poncho/split flap (27.9%) and rotation flap (2.9%). In 25% of the cases, exposures of the meshes were documented. Within this exposure rate, most of them were slight and only punctual (A = 16.2%), like one tooth width (B = 1.5%) and complete (C = 7.4%). A®- PRF provided significantly less exposures of the titanium meshes (76.5% no exposure vs. 23.5% yes, *p* = 0.029). Other parameters like tobacco abuse (*p* = 0.669), diabetes (*p* = 0.568) or surgical parameters (mesh size, defect region, flap design) did not influence the exposure rate. Surgical splints were not evaluated to reduce the exposure rate (*p* = 0.239). Gender (female) was significantly associated with less exposure rate (78,4% female vs. 21.6% male, *p* = 0.043).

**Conclusions:**

The results of this study suggest that the new digital protocol including patient-specific titanium meshes, resorbable membranes and bone grafting materials was proven to be a promising technique. To improve soft tissue healing, especially A®-PRF should be recommended.

## Background

Guided Bone Regeneration (GBR) technique offers the possibility to reconstruct bony alveolar defects [1] preceding implant placement [2]. A barrier membrane separates the surrounding connective tissue from the bony defect [3–7].

Large defects of the jaws exhibit both hard and soft tissue shortages. Titanium meshes are well-known to work as a mechanical scaffold and create stability for bone healing in large three-dimensional defects [8]. Therefore, rebuilding such a complex defect also implicates a detailed focus on soft tissue management. Membrane and graft exposures are frequent complications associated with non-resorbable membranes [9–12].

Based on the principles of the GBR technique, individualized titanium meshes are proposed to overcome the problems of the conventional titanium meshes [13]. Literature [14–16] reveals the advantages of this technology such as a shortened and facilitated surgery time in sense of a modern digital work-flow. Although Sumida et al. evaluated less exposures, but not statistically significant, in patient-specific titanium meshes [17], soft tissue management remains one of the most challenging targets in customized bone regeneration [18].

Treatment opportunities like self-inflating soft tissue expanders [19] or free fat grafts (FFG) from the buccal fat pad [20] aiming to stabilize the soft tissue healing process increase comorbidity of the patient and need additional surgical skills. A promising solution may be the enhancement of soft tissue wound healing by Platelet-Rich Fibrin (PRF) as shown in literature [21–23].

So far, there is no clear recommendation in literature to reduce this exposure rate in customized bone regeneration.

The aim of this study was to describe a new surgical protocol in customized bone regeneration and to evaluate parameters that minimize the risk of customized titanium mesh exposures. The influence of various demographic, local and systematic factors were assessed.

## Methods

This is a clinical non-interventional monocentre study. It was performed retrospectively during the clinical routine without any further consequences for the patient. Data were anonymized and processed in accordance with the 2013 Declaration of Helsinki on medical protocol and ethics (Declaration of Taipei on Ethical Considerations regarding Health Databases and Biobanks 2016). Due to the character of the study no approval by the local ethics committee was necessary (Regulatory of the ethic committee of Rhineland-Palatinate and described in Gaus et al. [24]). No administrative permissions and/or licenses were acquired by the team to access the data used in the research due to the character of the study.

### Study population

This study included 55 patients with 68 grafting procedures for consecutive dental implant placement. Patients with three-dimensional bony defects were included. All three-dimensional grafting procedures had to be performed by the same trained surgeon (MS; Private Dental Practice, Filderstadt, Germany) by using a patient-specific titanium lattice structure (Yxoss CBR®, ReOss, Filderstadt, Germany). Female and male patients > 18 years were included.

Exclusion criteria were mentally disabled patients, pregnant women and patients < 18 years. Local exclusion criteria were horizontal or vertical bony defects. Patients with three-dimensional defects treated with other bone augmentation procedures like distraction osteogenesis, block graft or onlay-technique were excluded as well. In general, patients with systemic or local diseases and malignancies were excluded before enrolment.

### Workflow and surgery

**Fig. 1:**
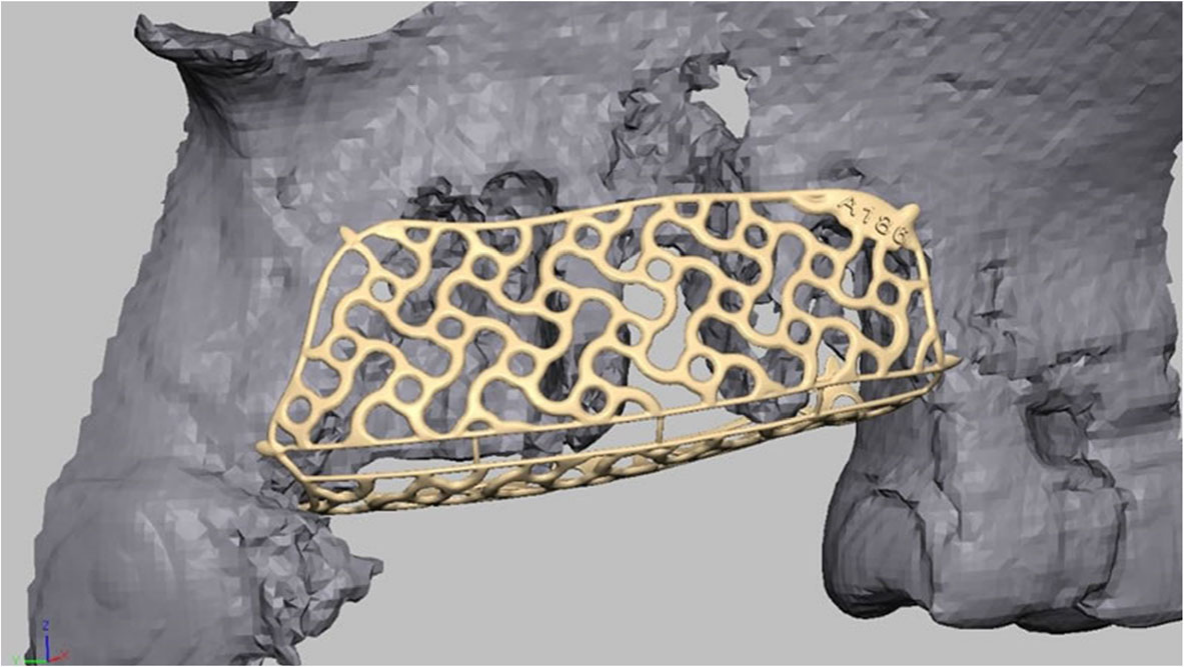
Design-example. The inner contour of the mesh represents the desired augmentation volume

**Fig. 2:**
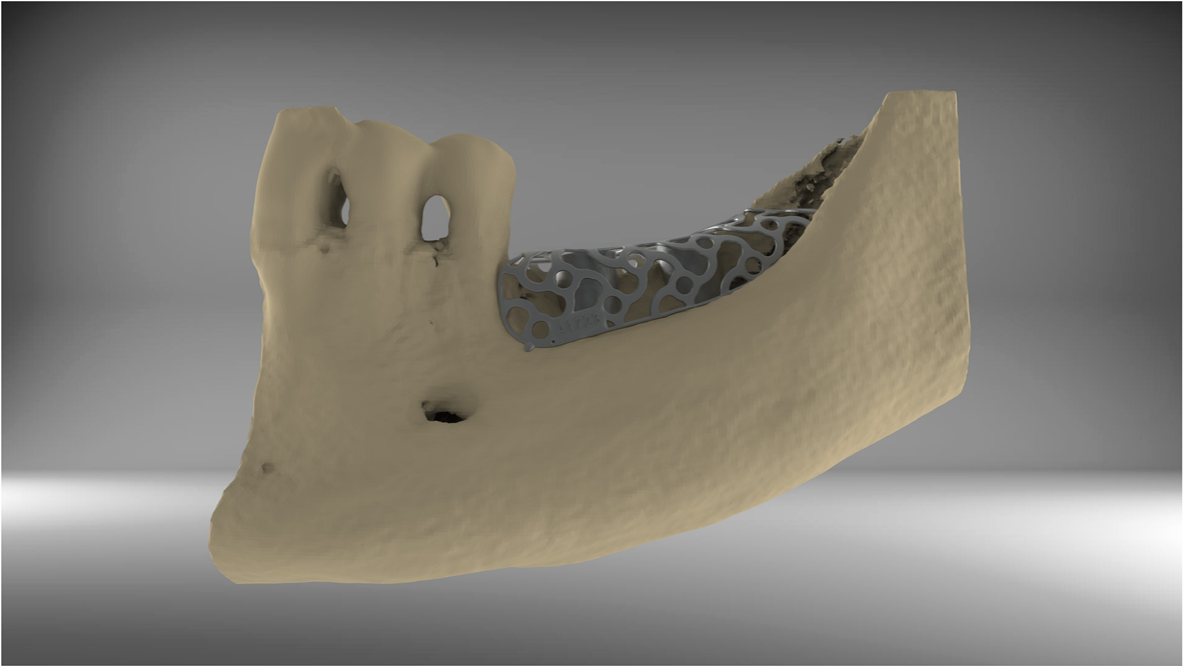
By using CAD/CAM-technology, the technician is able to design an individualized titanium mesh

**Fig. 3:**
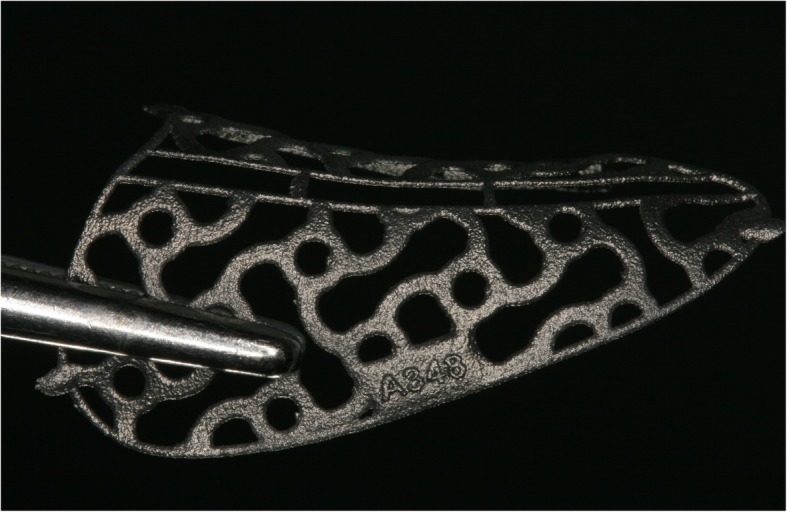
After an additive 3-D- printing process

After acquisition of a Cone Beam Computed Tomography (CBCT) dataset, a 3D-projection of the atrophied segment was obtained by using a reverse engineering software. The necessary bone volume was digitally added, and the individualized titanium meshes were designed. The inner contour of the lattice structure represented the desired augmentation volume. By using Computer-Aided Design/Computer-Aided Manufacturing (CAD/CAM) procedures and rapid prototyping the final design was achieved and confirmed interactively by the surgeon (Figs. 1 and 2). After a 3D-Printing Process (Fig. 3), the titanium mesh was sent to the surgeon and sterilized before use.

**Fig. 4:**
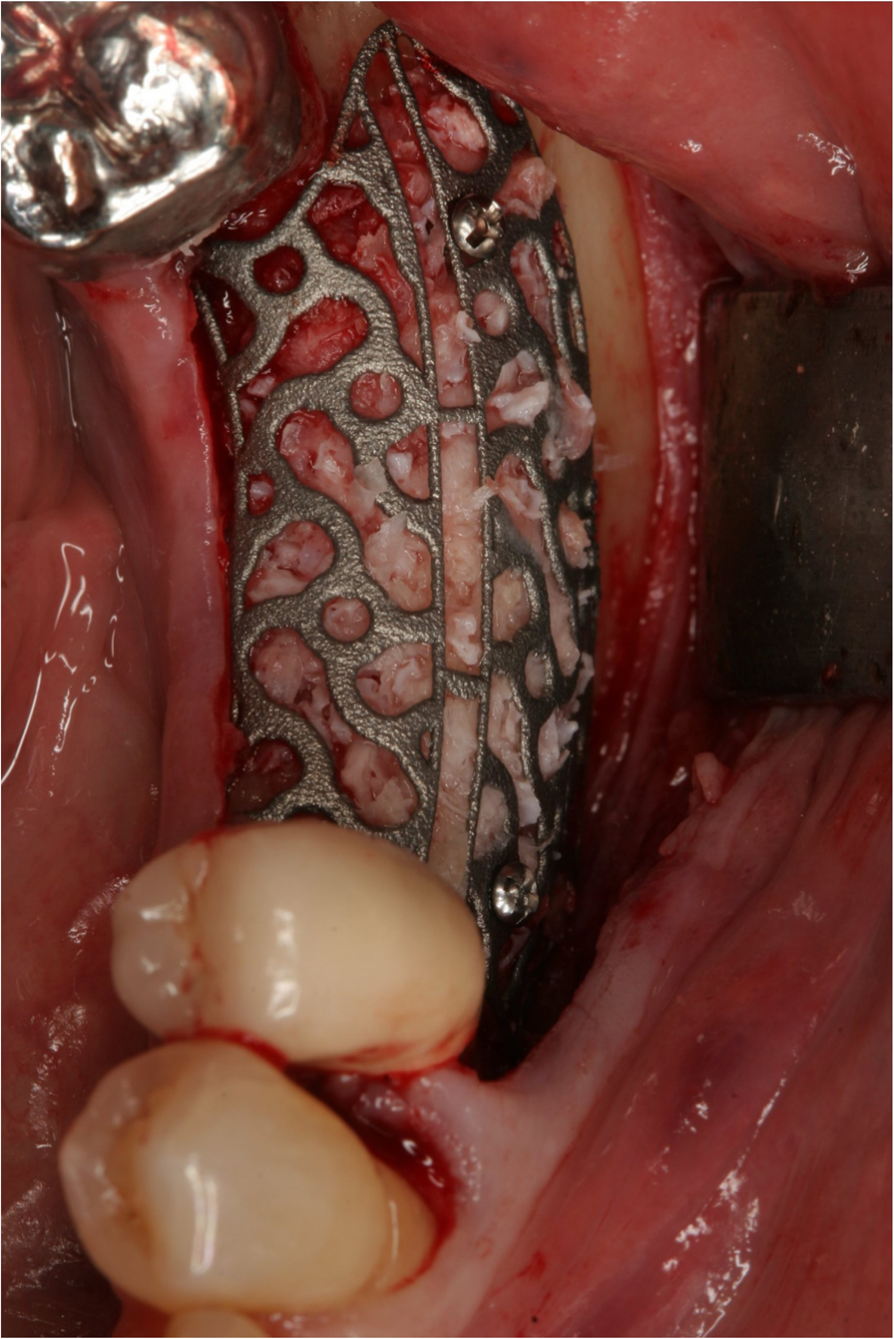
Individualized mesh in situ. Titanium mesh filled with graft; there is a slot for the easy removal function on top and central

**Fig. 5:**
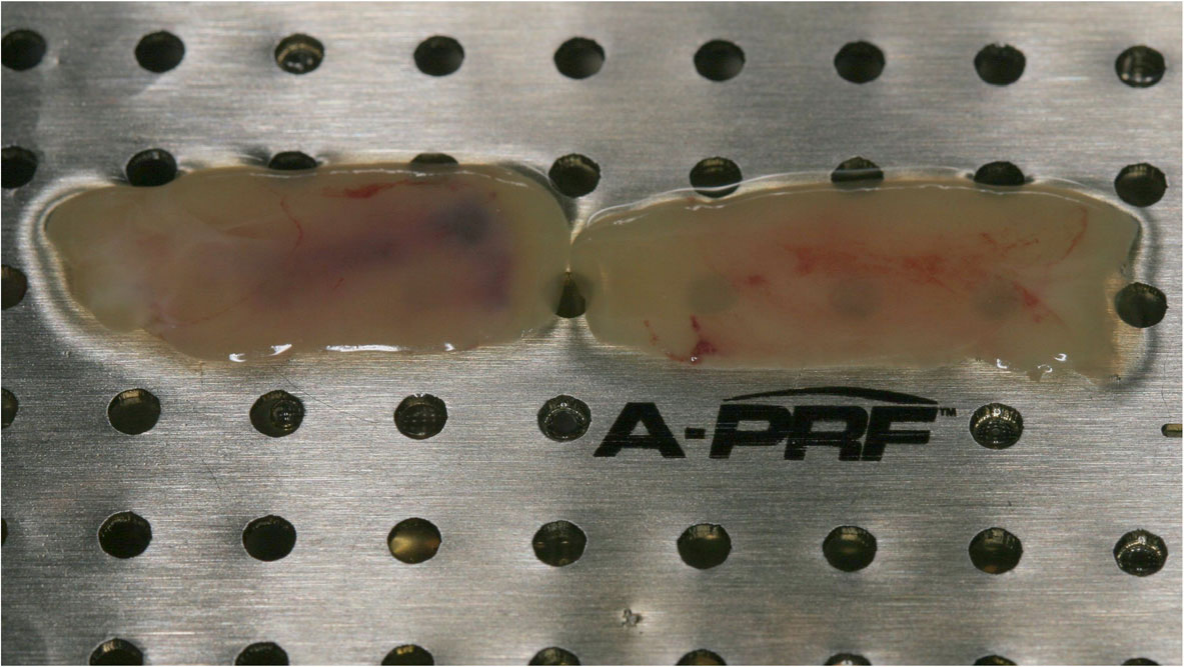
The A-PRF clots according to the protocol of Choukroun

**Fig. 6:**
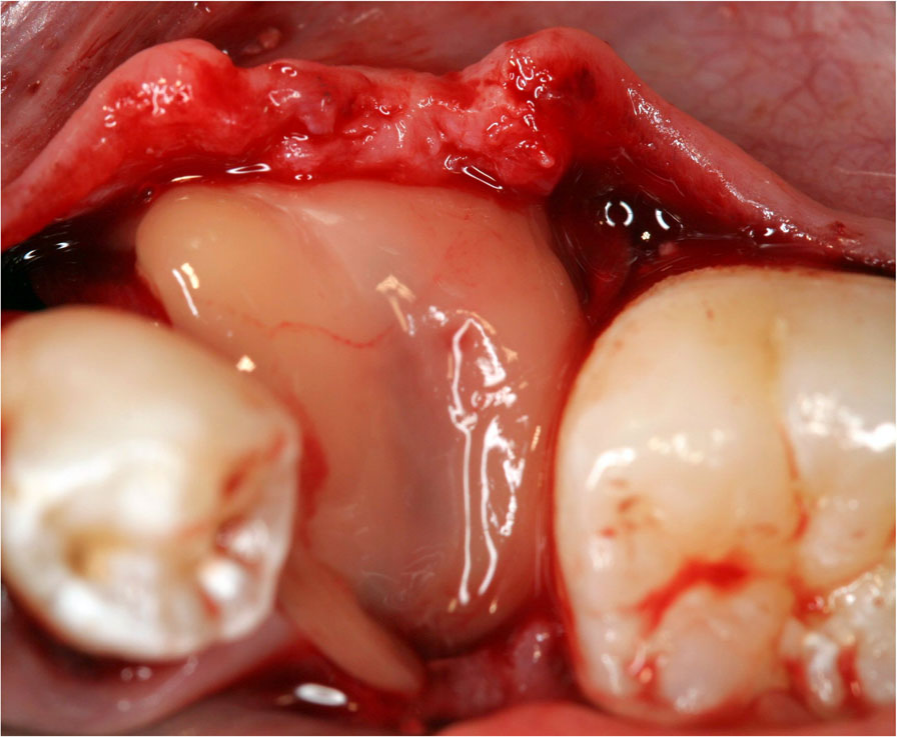
A-PRF clot covering a titanium mesh in situ

**Fig. 7:**
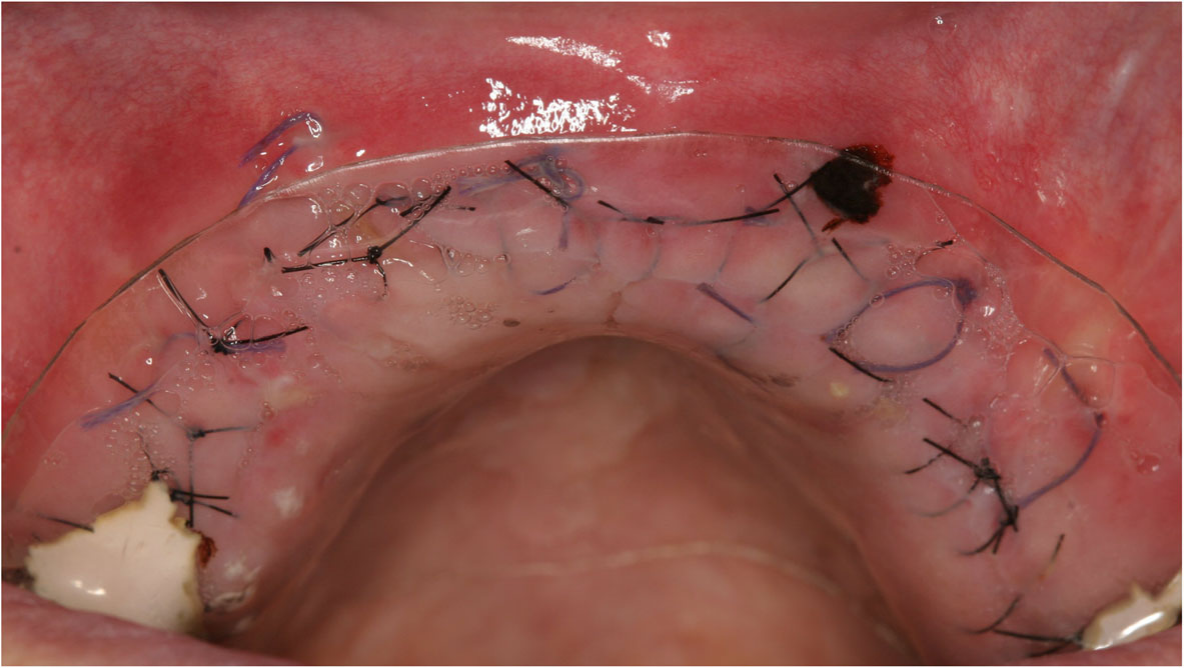
Surgical splint application after suturing without tension and a healing process of 10 days

Surgery was performed under local anesthesia. The opening incision was carried out in accordance with the defect size and location of the neighboring anatomical structures of the region. Flap design was performed as appropriate in each case (full flap preparation, full flap and periosteal incision, periosteal incision, poncho/split flap or rotation flap). After preparation of the defect, scar tissue was removed. In the lower jaw, some cortex perforations were performed to boost the blood supply. The meshes were installed by using a mixture of autologous bone graft and bone substitute biomaterial (Bio Oss®, Geistlich, Wolhusen, Switzerland) in a 1:1 ratio (Fig. 4). Autologous bone was harvested from the conventional intraoral donor sites (*n* = 59, external oblique line (*n* = 50) and operation site (*n* = 9)) or from the iliac crest (*n* = 8). In one patient, only bone substitute (Bio Oss®) was used. Each mesh was fixed to the residual bone with titanium osteosynthesis screws. A resorbable membrane (Bio-Gide®, Geistlich, Wolhusen, Switzerland) was placed on top. In some cases (*n* = 12), an Advanced-Platelet Rich Fibrin (A-PRF®) clot was applied according to manufacturers´ protocol following the in vitro-protocol of Choukroun [25] (Figs. 5 and 6). Flaps were adopted without tension by using deep mattress and single interrupted sutures (Seralon5/0). Vacuum form splints were adjusted to enhance soft tissue healing in *n* = 22 (Fig. 7). Patients received instructions concerning a proper oral hygiene. They had to avoid brushing at the grafting area and to wear removable dentures. All patients underwent an oral antibiotic therapy (Amoxicillin® 1000 mg 1–1-1 or Clindamycin® 600 mg 1–1-1) for 5–7 days starting at time of the surgery.

After a two-week healing period, sutures were removed. Outcomes were also assessed one week after surgery and during follow-up each month. In summary, they were controlled each month for 4 to 8 months depending on the defect. The Re-opening and removal of the titanium mesh was after approximately 4–8 months depending on size of the defect. Implant placement subgroups (Camlog Screw Line®, Camlog, Wimsheim, Germany) were equally distributed (implantation performed either simultaneously with mesh insertion (44.1%) or after a healing period of 4–8 months combined with the removal of the mesh (44.1%)).

### Outcome assessment

Primary outcome was the grafting success defined as the feasibility of implant placement in the planned position and achievement of an adequate primary stability (15Ncm-35Ncm) until the re-entry and to finalize with the individual prosthetic supraconstruction. Failure was defined as complete loss of the graft. Outcomes and possible healing difficulties were assessed one week after surgery and during follow-up each month. Patients were instructed to contact the surgeon if any disturbances occurred.

Secondary aim of the study was to assess possible risk factors (defect regions, defect and mesh sizes, smoking, tissue phenotype (thin and fragile phenotype, thick phenotype [26]), diabetes) for grafting success and developing an exposure. Exposure rate and impact of such factors as Vacuum form splint, A-PRF® and flap design on the exposure rate should be assessed. Exposures of the titanium mesh were classified concerning their size. “A” was a punctual exposure, “B” an exposure like one premolar size and “C” a complete one whereas “D” was no exposure [27]. Mesh size was defined according to the missing teeth.

### Data evaluation

Statistical assessment was done using IBM SPSS® Statistics version 22.0 for Windows. Level of significance was set to *p* < 0.05. Data presentation was performed by using JMP® 10.0 statistical software (SAS Institute, Cary, NC, USA). Grafting success, exposure rate and impact of such factors as Vacuum form splint, A-PRF® and flap design on the exposure rate.

For secondary outcome parameters possible risk factors (defect regions, defect and mesh sizes, smoking, tissue phenotype (thin and fragile phenotype, thick phenotype [25]), diabetes) for developing an exposure were defined. Statistical analyses were performed using Chi-Quadrat-Test and Fisher’s Exact-Test as appropriate for qualitative parameters, T-Test or Mann-Whitney-U-Test for quantitative parameters.

## Results

A group of 38 female (69.1%) and 17 male (30.9%) patients with 68 three-dimensional defects and a mean age of 58.1 (range of age 18 to 81 years with SD = ±15.6 years) were enrolled. Augmentation site was in the upper jaw (*n* = 35, 51.5%) and in the lower jaw (*n* = 33, 48.5%). No case failed. In total, 98 implants could be placed as planned. Yxoss CBR® was removed after mean time of 6.53 ± 2.7 months. Tobacco abuse was documented in *n* = 6 (10.9%) and stable diabetes mellitus (HbA1c < 5%) in n = 3 (5.5%).

Bone quality was found to be D1 (*n* = 12, 17.6%), D2 (*n* = 36, 52.9%), D3 (*n* = 13, 19.1%) and D4 (*n* = 7, 10.3%) according to Misch’s classification. Tissue Phenotype was classified as “thin and fragile” (*n* = 53, 77.9%) and “thick” (*n* = 15, 22.1%). Flap design was performed as full flap preparation (*n* = 19, 27.9%), full flap and periosteal incision (*n* = 27, 39.7%), periosteal incision (*n* = 1, 1.5%), poncho/split flap (*n* = 19, 27.9%) and rotation flap (*n* = 2, 2.9%). Implant placement was performed either simultaneously with mesh insertion (*n* = 30, 44.1%) or after a healing period of 4–8 months (*n* = 30, 44.1%). In *n* = 8 (11.8%) implant placement was not performed while data evaluation. A surgical splint was used in *n* = 22 (32.4%) and A®- PRF in *n* = 12 (17.6%).

In 25% of the cases (*n* = 17), exposures of the meshes were documented. Within this exposure rate, most of them were slight and only punctual (A = 16.2%, *n* = 11). Exposure like one tooth width (B = 1.5%, n = 1) and a complete (C = 7.4%, *n* = 5) occurred as well. Associated with these exposures, no loss of grafting material (86.8%, *n* = 59), partial (11.8%, *n* = 8) and complete in 1.5% (one case) was evaluated. A therapy according to the planned treatment protocol was possible in all the cases.

**Fig. 8:**
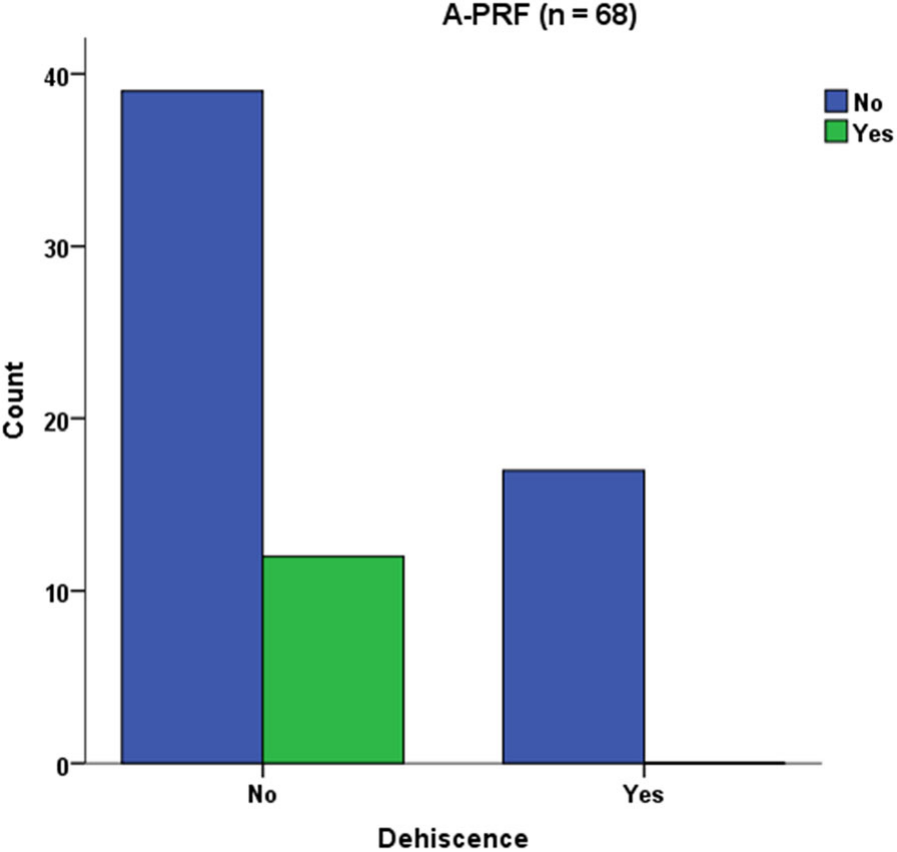
A®-PRF provided significantly less exposures of the titanium meshes

**Fig. 9:**
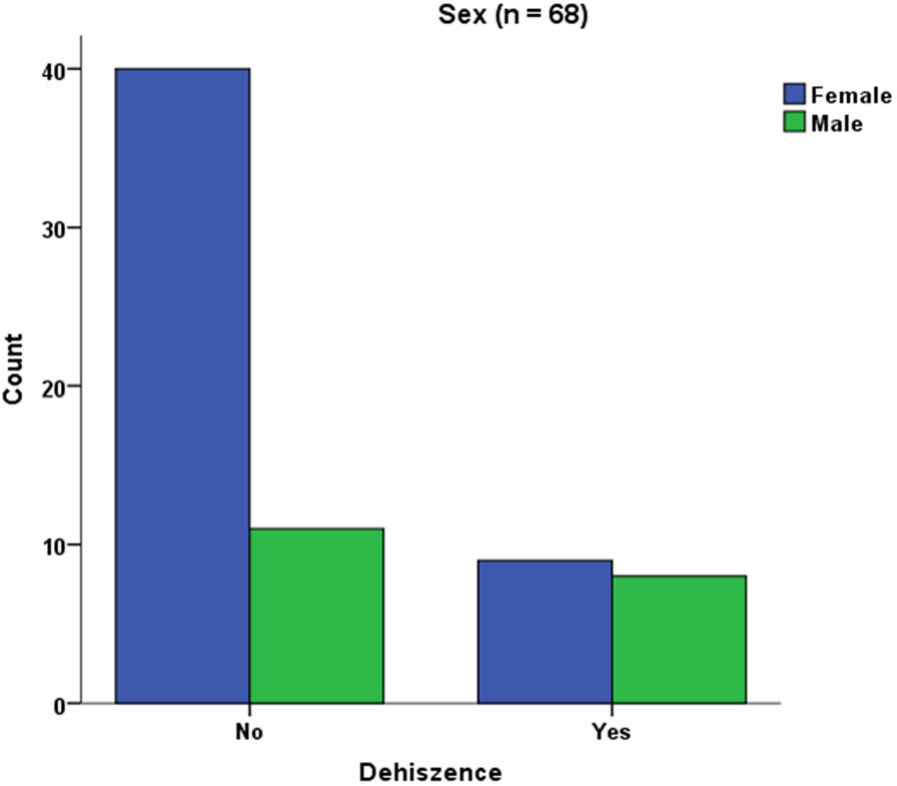
In female patients, significantly less exposures were considered

A®-PRF provided significantly less exposures of the titanium meshes (76.5% no exposure vs. 23.5% yes, *p* = 0.029) (Fig. 8). Other parameters like tobacco abuse (*p* = 0.669), diabetes (*p* = 0.568) or surgical parameters (mesh size, defect region, flap design *p* = 0.368) did not influence the exposure rate. Surgical splints were not found to reduce the exposure rate (*p* = 0.239). Gender (female) was significantly associated with less exposure rate (78,4% female vs. 21.6% male, *p* = 0.043, Fig. 9).

## Discussion

This study shows that treatment with customized titanium meshes offers the opportunity to provide high-quality work in large three-dimensional bony defects. The benefits like precise fit, shorter time of surgery, predictable outcome and good acceptance of the surgical procedure were already described in recent literature [27, 28]. Although exposures are a common complication associated with titanium mesh technique, grafting outcome was not affected. This is according to literature where exposure does not necessarily compromise the final treatment outcome [29–31]. Literature aims to reduce exposure rates. Interpreting the results from the present study (25% exposure rate), all patients presented large defects which would have made a conventional GBR technique with resorbable membranes impossible and would have required block augmentation from other intra- or extraoral donor sites, onlay technqiues or distraction osteogenesis.

Comparing mesh exposure (A-C) of this study to literature, heterogenous data are documented. In customized bone regeneration, Sagheb et al. reported 33% exposures [16]. Other studies range from uneventful healing with no [32–34] and from 14.8 to 59% [29, 30, 35–38] of membrane exposure. These different results may be caused by a lack in literature to precisely describe the exposures according to size. By distinguishing in severity of the exposure [27], we were able to show that most of them are only slight and only punctual. Another point is the various titanium mesh techniques, various surgical protocols and skills. Sumida et al. evaluated less exposure rates for customized meshes compared to the conventional titanium mesh technique [17]. Evaluating the grafting protocol without a control group is a shortcoming of this study due to its retrospective character. In general, there are two major limitations in this study that could be addressed in future research. First, the study focused on augmentation procedures in three-dimensional defects. No measurements of the defect (in mm^3^) were assessed. A more objective way to describe them clinically and radiographically by CBCT would be in mm^3^. This shortcoming is due to the retrospective character of the study and the next prospective study will define the three-dimensional defects in mm^3^ and according to a new classification.

Second, grafting outcome should be defined in mm^3^ after another CBCT and matching the two radiographs. According to our best knowledge, and lots of own tests, matching will not work exactly because of X-ray scattered radiation of the titanium lattice structure. Future research should aim to develop a new software excluding these effects.

Within the limitations of this study being retrospective without a control group it was possible to show that an exposure does not necessarily lead to grafting failure as defined above. A prospective study should compare different methods for bone augmentation in similar defect sizes in comparable study groups to present a superiority of one method.

Analyzing risk factors for soft tissue healing, the results of this study show that tobacco abuse had no influence on the exposure rate. This is surprising because many studies describe the negative effects of tobacco abuse in healing processes because it hinders revascularization and enforces soft tissue inflammation [30] [39]. Our findings are supported by Lindfors et al. [30], who stated no correlation between smoking and the development of exposures. The results may be due to a proper informed consent of the patients directly and their acceptance to reduce/avoid smoking after surgery. Additionally, the results may also be associated with the limited number of patient subgroups in this study.

The same goes for the findings concerning Diabetes. Diabetes as another risk factor in wound healing processes [40] was not proven to have an influence on developing exposures. It is tempting to speculate that all the included patients suffering from diabetes are well-controlled type 2 diabetic patients and Erdogan et al. [41] have found GBR technique in such cases being a proper treatment opportunity.

One might assume that a thin tissue phenotype [26, 42] is much more difficult to handle than a thick one. Scar tissue will develop easily because of an earlier mucosal rupture. An adequate aesthetic outcome may be difficult to achieve. This was evaluated in previous studies [43]. They described thick gingival phenotypes associated with additional blood support during wound healing because of the missing periodontal ligament support in implant therapies. These findings are contrary to the results at hand. A possible reason for this may be in the patient group itself. Large three-dimensional defects like described in this study, are the result of numerous preceding surgeries like teeth extraction, inflammation processes or others. According to authors opinion, scar tissue as a result of these former interventions – in thin and thick tissue phenotype – might be the real reason for developing an exposure.

In this study, neither age nor periodontitis influence the exposure rate. These findings are according to Sagheb et al. [16].

In their study, they also evaluated no relationship between gender and the risk to develop an exposure. This goes along with other studies [44] but is not in accordance with findings of this study where we were able to find a significant relationship between male gender and the development of exposures. Male patients were already described to suffer from an increased potential infection rate after implant placement by Figueiredo et al. [45]. This is in accordance with Kim et al. [46] who evaluated male gender to have the highest risk of wound dehiscence in guided bone regeneration. These results are in line with our findings. The same observation was also described in dermatology. Dao et al. [47] reported about gender differences in skin regeneration. They described modified immunological processes in elderly men caused by a decreasing testosterone level. Male-specific instructions on postoperative care may help to overcome these healing difficulties. On the other hand one might assume that especially steroid hormones in female patients appear capable of influencing the normal bacterial flora and the subgingival ecology [48, 49]. Consecutively, healing difficulties may arise. This is not according to the results of this study. Female gender was significantly associated with less exposures. Further gender-specific studies in intraoral healing processes are needed.

Localization or sizes of meshes were not relevant for the occurrence of dehiscence. This is contrary to the findings of Uehara et al. [50], wo found a significant correlation between the success rate of a bone grafting and the extension of the augmentation site. This may be due to different techniques in flap management or other patient recruitment. In the study at hand, there were no differences between the upper and lower jaw concerning an exposure rate as already described in Her et al. [31] and Louis et al. [37].

Although flap design did not affect the outcome of the treatment, according to authors´ opinion, a proper flap management will remain a key point in working with individualized titanium meshes. This includes planning of the incision in advance according to Kleinheinz et al. [51] and adopted to the kind of defect, a careful flap elevation and a tension-free primary wound closure [11]. This will avoid necrosis of the flap and a premature exposure. The risk of a consecutive inflammation and possible loss of the graft will be decreased [10, 52].

This study evaluated a significant improvement of therapy outcome if customized titanium mesh technique was applied together with A-PRF®. This is according to a recent study which found the combination of PRF and FFG to be successful during the soft tissue healing process preceding implant placement [53]. Torres et al. [54] also reported 28.5% of their cases in a control group suffering from mesh exposure while in a PRP group, no exposures were registered. The success of PRF may be due to the role of fibrin in initial clot stabilization [22]. PRF as a biodegradable scaffold consisting of stem cells, fibrin, platelets and leucocytes boosts microvascularization and epithelial cell migration [55, 56]. This may prevent mesh exposure by using it to cover meshes as applied in this study. In a recent systematic review, there was limited evidence on the effects of Lykocite Platelet Rich Fibrin (L-PRF) in intraoral bone grafting procedures [55]. They concluded the need for further studies with special emphasis on the standardized surgical procedures [55]. Therefore, and supported by this study, a proper soft tissue healing is boosted using (A®-) PRF®. The influence on bone healing processes must be assessed in further randomized, control studies, although a recent study described positive effects in post-extraction sockets [23].

The use of surgical splints must be evaluated in prospective studies with more patients. According to authors opinion, these surgical splints may provide a better wound healing although the results being not significant in this study. As a part of everyday routine together with soft tissue surgery, it is not a standard procedure applied together with customized mesh technique or titanium meshes in general. Another study agreed that – especially in male patients – the application of surgical pack and surgical splint would provide a better wound healing [46].

## Conclusion

In complex bone reconstruction, the new surgical protocol in customized bone regeneration was proven to be a promising technique.

Interpreting the primary outcome of this study, exposures are proven to be complications that did not affect the defined outcome and success of the grafting procedure. To improve soft tissue healing, especially A®- PRF should be recommended. A tension-free wound closure seems to be more important than a specific flap design. Concerning the secondary aim of the study, future prospective research should aim to evaluate the gender specific risk of developing exposures and in general for developing healing difficulties in augmentation procedures.

## Data Availability

The datasets used and/or analysed during the current study are available from the corresponding author on reasonable request.

## Abbreviations

3D-Printing Process: 3Dimensional-Printing Process
3D-projection: 3Dimensional-projection
A-PRF®: Advanced-Platelet Rich Fibrin
CAD/CAM: Computer-Aided Design/Computer-Aided Manufacturing
CBCT: Cone Beam Computed Tomography
CBR®: Customized Bone Regeneration
FFG: free fat grafts
GBR: Guided Bone Regeneration
IBM SPSS®: Statistics software
JMP®: Statistics software
L-PRF: Lykocite Platelet Rich Fibrin
Mann-Whitney-U-Test: Statistical test
mm3: cubic millimetres
MS: Marcus Seiler
N: number
PRF: Platelet-Rich Fibrin
T-Test: Statistical test
